# Performance of early- and intermediate-maturing maize hybrids for grain yield and secondary traits under multiple stress environments

**DOI:** 10.3389/fpls.2026.1808200

**Published:** 2026-07-24

**Authors:** Lucia Z. Ndlala, Admire I. T. Shayanowakho, Takudzwa Mandizvo, Julia Sibiya, Kingstone Mashingaidze, Jill Cairns

**Affiliations:** 1 Agricultural Research Council-Grain Crops Institute, Potchefstroom, Northwest, South Africa; 2 School of Agricultural, Earth and Environmental Science, University of KwaZulu-Natal, Pietermaritzburg, South Africa; 3 International Maize and Wheat Improvement Center (CIMMYT), World Agroforestry Centre (ICRAF), Nairobi, Kenya; 4 Global Maize Program, International Maize and Wheat Improvement Center (CIMMYT), Harare, Zimbabwe

**Keywords:** maize hybrids, multiple stress tolerance, multivariate analysis, secondary traits, trait associations

## Abstract

**Introduction:**

Maize productivity in Southern Africa remains below global averages due to multiple stress factor.

**Methods:**

This study evaluated 87 early-and intermediate-maturing maize hybrids across 13 environments (stress and non-stress) using an alpha lattie design.

**Results:**

Significant variation (P<0.05) was observed. Grain yield ranged from 1.41 to 7.43 t ha^-1^ across stress conditions. Ears per plant and ear height re key contributors to yield.

**Discussions:**

Multivariate analysis identified 20 high-perofimng, stable hybrids suitable for multi-environment testing.

## 1 Introduction

Maize (*Zea mays* L.) is a staple crop and a cornerstone of food security in Southern Africa, providing a significant share of the region’s caloric intake and agricultural output. Across East and Southern Africa, cereals account for approximately 19.7% of daily caloric intake, underscoring their critical role in food security and economic stability. In South Africa (SA), maize dominates grain production, accounting for over 70% of total output and covering more than 60% of the country’s arable land (Duffy and Masere, 2015; Tesfaye et al., 2015).

South Africa is the leading maize producer in the Southern African Development Community (SADC), with an average annual productivity of 9 t ha^-1^. The country contributes nearly 50% of the region’s maize output, with 58% of its cropping area devoted to maize production (Adisa et al., 2018). Approximately 25% is exported to neighboring countries, reinforcing maize’s role in regional trade (Geyser et al., 2024; Mapfumo et al., 2020). While commercial farms account for 85% of total maize output, smallholder and subsistence farmers contribute 15% (Nji et al., 2022). Other key producers in the region include Zambia, where subsidized inputs have boosted smallholder productivity (Gbegbelegbe et al., 2024), and Malawi, where the Farm Input Subsidy Program (FISP) has significantly increased maize yields (Fisher et al., 2015).

Despite its regional significance, maize production is challenged by climate variability. Changes in rainfall patterns, rising temperatures, and extreme weather events, such as the severe 2015 drought, have led to substantial yield losses in SA (Simanjuntak et al., 2023). Research indicates that an increase of 1 °C in temperature can reduce maize yields by 1% under optimal conditions and up to 1.7% under drought stress (Shiferaw et al., 2011). Higher temperatures also accelerate evapotranspiration, compounding water stress during critical growth stages (Cairns et al., 2013). In addition to climatic pressures, low soil fertility limits yield potential and maize resilience (Mitiku et al., 2024; Mwanza et al., 2020). Biotic stressors, particularly pests and diseases, are also intensifying due to climate change. Rising temperatures and altered rainfall regimes influence pest life cycles and weaken host resistance, increasing the frequency and severity of outbreaks (Keno et al., 2018; Njeru et al., 2023). Addressing these challenges requires breeding maize varieties with enhanced resistance to abiotic and biotic stresses.

Institutions such as the International Maize and Wheat Improvement Center (CIMMYT) and the International Institute of Tropical Agriculture (IITA) have developed stress-tolerant maize varieties tailored for sub-Saharan Africa (SSA). These breeding programs have produced hybrids and open-pollinated varieties (OPVs) with improved tolerance to drought, heat, and low N conditions (Cairns et al., 2013; Dao et al., 2015; Suwarno et al., 2014). The two centers have a reported annual yield gains of 0.85%–2.2% in hybrid and OPV pipelines over the past decade (Masuka et al., 2017; Tarekegne et al., 2023). These advances highlight the success of strategic breeding using diverse germplasm and heterotic groupings.

Evaluating maize hybrids under multiple-stress conditions is crucial for developing resilient varieties suited to Southern Africa’s variable environments. However, grain yield alone is not always a reliable indicator of performance under stress. This is because it is a complex, integrative trait influenced by numerous physiological and morphological processes, many of which are differently affected by specific stressors such as drought, heat, or low N. Under such conditions, yield may not accurately reflect a genotype’s adaptive potentials or resilience mechanisms.

Therefore, understanding the association among secondary traits, such as anthesis days, anthesis–silking interval, ears per plant, and ear and plant height, is important. These traits often show a more stable expression under stress and serve as indicators of stress tolerance. Knowledge of these traits’ interactions with grain yield under adverse conditions enables breeders to select for genotypes with more robust stress-adaptive profiles, even when yield data is confounded by environmental variability. Therefore, the objective of this study was to determine the association between grain yield and its secondary component traits across diverse stress conditions to guide the selection of multiple-stress-tolerant hybrids.

## 2 Materials and methods

### 2.1 Germplasm

A total of 87 maize hybrids, comprising 52 early-maturing and 35 three-way intermediate hybrids (each including checks), were evaluated in a single field experiment during the 2019/2020 planting season in South Africa and Zimbabwe under stress and non-stress environments. The CIMMYT inbred lines were selected specifically for their resilience against various biotic and abiotic stresses. The detailed description of the hybrids used in this study is presented in **Supplementary Table 1**.

### 2.2 Experimental design and field management

The experiments were laid out in a 3 × 29 alpha lattice design with two replications. Entries were planted in two-row plots that were 4 m in length, with 0.75 m between rows and 0.25 m within rows. Two seeds were planted and thinned to one plant per hill, resulting in a final population density of 53,333 plants ha^-1^ after a few weeks of germination. Two border rows were planted around each experiment to reduce the border effect. Standard agronomic practices for maize were applied at all sites. Gramoxone, Dual, Basagran, and 2,4-D herbicides were applied to control weeds, along with occasional manual weeding. Pest and disease management strategies were applied in accordance with local recommendations.

The experimental sites for the non-stress treatment were supplied with all the recommended fertilizers and irrigation when needed. Basal fertilizer, NPK (Zn) 3:2:1 (25), was applied at planting at 125 kg ha^-1^ of N, 83 kg ha^-1^ of P, and 42 kg ha^-1^ of K, as recommended for the sites. In addition, 280 kg ha^-1^ of N was applied 4 weeks after emergence (WAE) as a top-dressing fertilizer. In a low-N environment, we established sites that had been continuously depleted of N for over 10 years through the monoculture of oats (*Avena sativa*) with complete crop residue removal after harvest. Such management is known to reduce available soil N to suboptimal levels for maize production and is commonly used for low-N screening in maize breeding.

**Figure 1:**
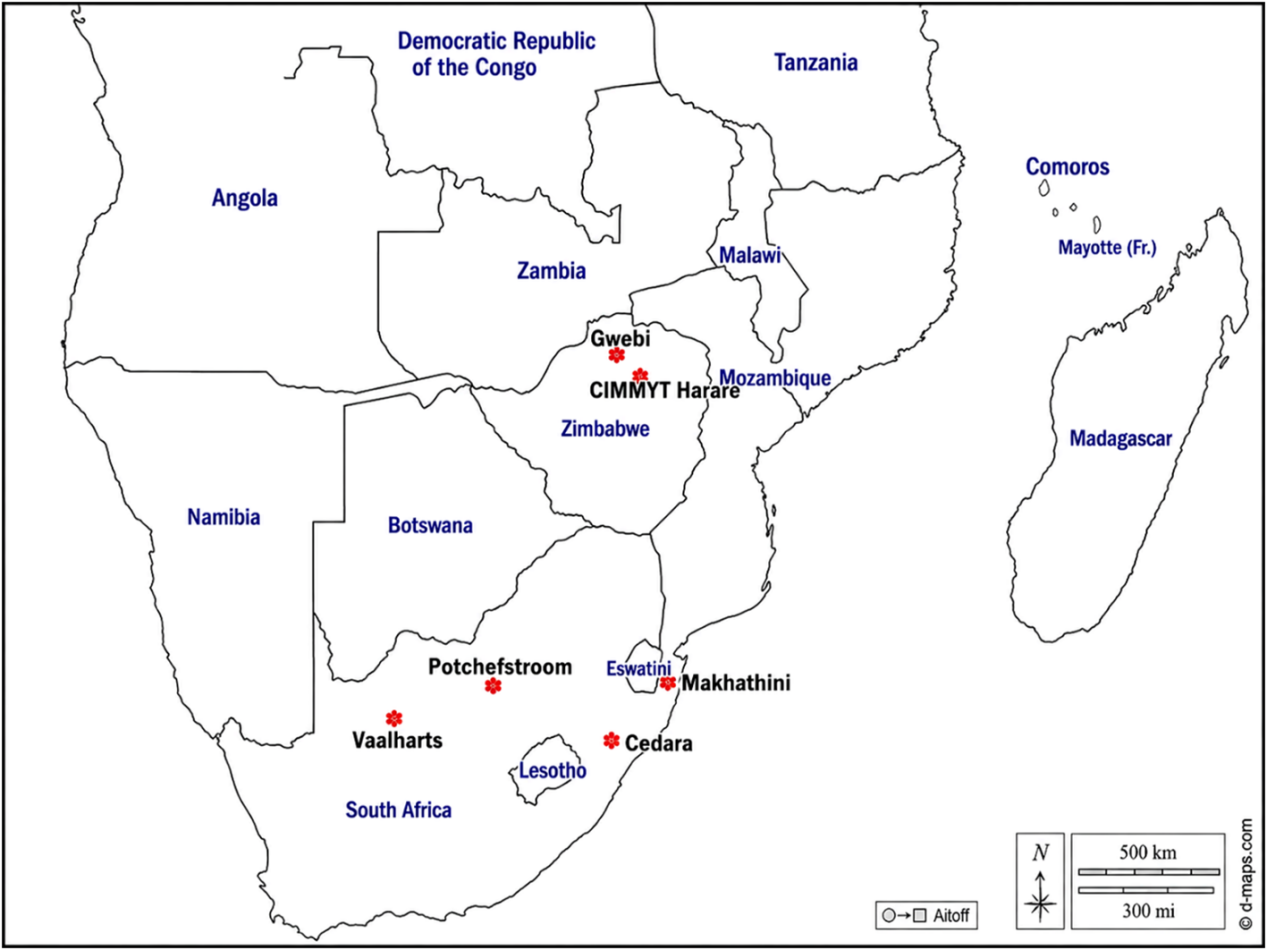
Geographic representation of the study locations.

The experimental sites for a managed drought stress trial were established during dry seasons, when stress was imposed at flowering and grain filling by stopping irrigation 2 to 3 weeks before anthesis and continuing until 4 weeks after anthesis (Bänziger et al., 2000). The experimental sites for heat-stress environments were planted during high heat, when maximum daily temperatures exceeded 35 °C during the flowering and grain-filling stages, consistent with thresholds reported to cause yield reduction in maize. The experimental sites for disease-stress trials were established at locations with natural disease pressure, dominated by foliar diseases typical of the region. Disease severity was sufficient to cause measurable yield reduction relative to non-stress trials. The geographic representation is presented in **Figure 1**, and the locations’ coordinates and management are described in **Table 1**.

**Table 1:** Description of coordinates and management of the locations used for evaluation in the 2019/2020 planting season.

Locations	Coordinates		Manag.	Envir.	Altitude	Temp. (°C)		Rainfall
	Lat.	Long.			(masl)	Max	Min	(mm)
Cedara	-29.5366	30.2664	Non-stress	E1	1,068	30.29	4.81	697.83
Cedara	-29.5358	30.2673	Disease	E2	1,068	30.29	4.81	697.83
Potchefstroom	-26.7304	27.0806	Non-stress	E3	1,349	25.81	11.31	363.8
Potchefstroom	-26.7342	27.0663	Low N	E4	1,349	25.81	11.31	363.8
Cedara	-29.5378	30.2661	Low N	E5	1,068	30.29	4.81	697.83
Makhathini	-27.4203	32.1935	Heat	E6	77	36.66	9.45	210.93
Makhathini	-27.40100	32.1839	Non-stress	E7	77	36.66	9.45	210.93
Makhathini	-27.4016	32.1834	M. drought	E8	77	36.66	9.45	210.93
Vaalharts	-28.0167	24.7166	Low N	E9	1,180	30.37	3.42	295.3
CIMMYT-Har.	-17.8	31.05	Non-stress	E10	1,489	31.67	10.85	321.68
CIMMYT-Har.	-17.8	31.05	Non-stress	E11	1,489	31.67	10.85	321.68
CIMMYT-Har.	-17.8	31.05	Non-stress	E12	1,489	31.67	10.85	321.68
Gwebi	-17.6833	30.8666	Non-stress	E13	1,406	36.4	8.82	358.6

Har., Harare; Lat., latitude; Long., longitude; Manag., management; Envir., environments; masl, meters above sea level; Max, maximum; Min, minimum.

### 2.3 Data collection

Data were recorded on anthesis days (AD), and silking days (SD) were measured as the days after planting when 50% of the plants per plot had shed pollen and 50% had silks, respectively. Anthesis–silking interval (ASI) was determined as the difference between days to silking and anthesis. Plant height (PH) and ear height (EH) (cm) were measured from five representative plants per plot after anthesis. The number of ears per plant (EPP) and ear position (EPO) were obtained as a ratio of the total number of ears divided by the total number of plants per plot and ear height divided by plant height, respectively. Grain yield (GY) was calculated using the grain weight per plot adjusted to 12.5% moisture content (Equation 1).

(1) $$\text{GY}=\;\frac{\text{Grain weight}}{1000}\;\times \;\left(\frac{100-\text{Grain moisture}\left(\%\right)}{100-12.5}\right)\times \frac{10}{\text{net plot area}}$$

### 2.4 Statistical analysis

#### 2.4.1 Analysis of variance

Analysis of variance (ANOVA) for single sites was performed on grain yield and its components, followed by a combined analysis of variance using GenStat software 21st Edition. Replications within environments were treated as random effects, while genotypes were considered fixed effects. Environments were considered random to allow inference across target production environments. The single-site ANOVA model used is given in Equation 2.

(2) $$Y_{ijk}=\;\mu +G_{i}+\;R_{j}+\;B_{k(j)}+\;{\text{ϵ}}_{ijk}$$

where *Y_ijk_* is the individual observation in each plot, *µ* is the grand mean for each variable, *Gi* is the effect of the *i*-th genotype (where *i* is 1, 2, 3…50), *R_j_* is the number of replications (where *j* is 1, 2), *B_k(j)_* is the estimate of the incomplete block within replication (where *k* is 1, 2), and *ε_ijk_* is the overall random error effect.

The combined ANOVA model used is shown in Equation 3.

(3) $$Y_{ijkl}=\mu +\;G_{i}+\;E_{j}+\;R_{k(j)}+B_{l(jk)}\;+\;GE_{ij}+\;GE_{ijr}+\;{\text{ϵ}}_{jilk}$$

where *Y_ijk_* is the response of the *i*-th genotype in *j*-th environment and *k*-th replication within environment and *l*-th block within replication, *µ* is the grand mean, *G_i_* is the genotype effect *i*, *E_j_* is the environment effect *j*, *R_k(j)_* is the replication within environment effect *k*, *B_l(jk)_* is the block within replication effect *l*, *GE_ij_* is the genotype × environment interaction effect, *GE_ijr_* is the region × genotype × environment interaction effect, and ε*_ijlk_* is the random error.

Traits BLUPs were computed using META-R (Alvarado et al., 2020).

#### 2.4.2 Principal component biplots, correlations, and hierarchical clustering

Principal component analysis (PCA) was analyzed in XLSTAT software (Lumivero, 2023). To visualize the relationship between genotypes and multiple traits, a genotype-by-trait (GT) biplot was constructed using the GGE biplot methodology described by Yan and Rajcan (2002). The biplots were generated using the standardized trait means derived from META-R, a statistical tool commonly used for multi-environment trial analysis. Pearson correlations and dendrograms based on the unweighted pair-group method of arithmetic clustering were computed on RStudio.

#### 2.4.3 Path coefficient analyses

The path coefficient was calculated using Microsoft Office Excel software by taking the Pearson phenotypic correlation data to determine each variable’s contribution (direct and indirect) to the total effect (Akintunde, 2012). Equation 4 was used to calculate the path coefficient:

(4) $$y=a+\;b_{1}\;X_{1}+\;b_{2}X_{2}\;+\;b_{3}\;X_{3}+\;U$$

where *y* is the single response variable (grain yield) and a + b_1_*X*_1_ + b_2_*X*_2_ + b_3_*X*_3_ + U are variables from correlation data with the assumption that the values of the variables are random, normally distributed and that the causal variables are independently contributing to the dependent variable (grain yield).

## 3 Results

### 3.1 Analyses of variance and mean performance

Analysis of variance under stress and non-stress environments showed significant differences in all of the traits recorded for the hybrids. In optimal environments, the interactions region × genotype, region × environment, region × genotype × environment, and genotype × environment were significant for grain yield and its secondary traits (**Table 2**). The anthesis days (AD) ranged from 73.22 to 85.29, with a mean of 78.63 (**Supplementary Table 2**). The anthesis–silking interval (ASI) ranged from -1 to 2, with a mean of 0.57. Plant height (PH) ranged from 189.76 to 231.66 cm, with a mean of 213.21 cm. Ear height (EH) ranged from 94.52 to 139.05 cm, with a mean of 116.79 cm. Ear position (EPO) ranged from 0.48 to 0.61, with a mean of 0.55. Ears per plant (EPP) ranged from 0.83 to 1.10, with a mean of 0.96.

**Table 2:** Mean sum of squares from combined analysis of variance of 87 early to mid-maturing maize hybrids under stress and non-stress conditions in two regions.

Sources of variation	d.f.	AD	ASI	EH	PH	EP		EPP	GY	
Non-stress										
Region (R)	1	1,372.57^***^	151.69^***^	194,450.19^***^	897,465.10^***^	0.06^***^		1.28^***^	1,698.14^***^	
Genotype (G)	86	135.22^***^	3.56^***^	1,114.93^***^	1,609.10^***^	0.01^***^		0.10^***^	5.91^***^	
R × G	86	15.34^***^	1.78^***^	189.13^***^	402.30^***^	0.00^*^		0.09^***^	1.46^***^	
R × environment (E)	6	33,432.81^***^	102.03^***^	26,578.49^***^	71,634.80^***^	0.06^***^		4.83^***^	1,080.29^***^	
R × G × E	429	8.59^***^	1.39^***^	131.17^***^	410.50^***^	0.00^**^		0.03^***^	1.64^***^	
E × Rep	8	2.68^*^	3.38^***^	820.92^***^	1,143.30^***^	0.01^***^		0.27^***^	11.45^***^	
E × Rep/Blk	216	6.77^***^	1.49^***^	211.87^***^	765.40^***^	0.00^**^		0.04^***^	2.07^***^	
G × E	429	6.94^***^	1.24^**^	98.94^**^	219.10^***^	0.00^*^		0.02^***^	1.25^***^	
Residual	385	2.81	1.00	78.14	126.40	0.00		0.02	0.82	
Low N										
Genotypes	86	45.04^***^	1.74^*^	351.20^***^	432.70^***^	0.00^***^		0.03^**^	1.34^***^	
Environments	2	22,749.97^***^	118.17^***^	105,990.75^***^	295,073.20^***^	0.22^***^		2.13^***^	22.07^***^	
Environments × Rep	3	71.78^***^	1.66^*^	2,053.73^***^	4,740.60^***^	0.01^**^		0.05^**^	5.39^***^	
Environments × Rep/Blk	90	29.97^***^	1.63^*^	378.39^***^	534.70^***^	0.00^***^		0.04^***^	1.62^***^	
Environments × genotypes	172	10.71^***^	1.38^*^	166.34^***^	215.70^***^	0.00^***^		0.03^**^	0.87^***^	
Residual	168	4.96	1.31	87.33	114.2	0.00		0.02	0.45	
Heat										
Rep	1	0.01	11.64^*^	328.28^**^	2,800.00^***^	0.00^*^		0.01^*^	0.13^*^	
Rep/Blk	30	24.77^*^	4.56^*^	235.75^***^	358.60^**^	0.01^**^		0.04^*^	2.39^***^	
Genotypes	86	18.95^*^	2.66	155.40^**^	167.70*	0.01^*^		0.03^*^	0.82^**^	
Residual	56	13.44	3.60	87.08	191.50	0.00		0.03	0.55	
Disease										
Rep	1	4.83^*^	1.13	72.70	20.10	0.00	0.00		0.13^*^
Rep/Blk	30	31.97^***^	0.75	303.60^***^	346.60^***^	0.00^**^	0.02^*^		2.74^***^
Genotypes	86	15.38^***^	0.99*	258.10^***^	270.70^***^	0.00^***^	0.02^*^		1.54^***^
Residual	56	3.43	1.04	106.3	121.5	0.00	0.01		0.37
Managed drought										
Rep	1	15.54^**^	0.09	704.02***	4,201.30^***^	0.00^*^	0.00		6.05^**^
Rep/Blk	30	14.13^***^	4.71**	662.82***	1,189.50^***^	0.02^***^	0.02^**^		3.03^***^
Genotypes	86	11.39^***^	3.06*	172.10***	425.20^**^	0.01^***^	0.02^*^		1.00^*^
Residual	56	3.34	2.75	59.80	233.60	0.00	0.01		0.73

d.f., degrees of freedom; Rep, replications; BLK, blocks; AD, anthesis days; ASI, anthesis–silking interval; EH, ear height; PH, plant height; EP, ear position; EPP, ears per plant; GY, grain yield.

*Significant at 0.5 probability level.

**Significant at 0.01 probability level.

***Significant at 0.001 probability level.

The mean grain yield performance for all of the environments is presented in **Supplementary Table 3**. The grain yield ranged from 3.77 to 5.23 t ha^-1^ across environments, 5.50 to 7.43 t ha^-1^ under non-stress, 2.11 to 3.36 t ha^-1^ under low N, 2.38 to 6.67 t ha^-1^ under disease, 1.41 to 3.35 t ha^-1^ under heat, and 2.93 to 4.02 t ha^-1^ under managed drought for early to intermediate hybrids. The mean grain yield of the top 10 performing genotypes for early-maturing hybrids ranged from 5.05 to 5.23 t ha^-1^ across environments (**Table 3**). Hybrid CML571/CML546//CZL15225-B showed the highest mean yields under low N (3.15 t ha^-1^) and managed drought (4.00 t ha^-1^) environments and showed the lowest ear height (91.29 cm) and ears per plant (0.95). Hybrid CML571/CML546//CZL15089 showed the lowest mean yields across environments (5.05 t h^-1^), optimum (7.01 t ha^-1^), and low N (2.82 t ha^-1^). However, the hybrid showed the highest mean yield under disease environment (6.66 t ha^-1^).

**Table 3:** Mean performance of early to mid-maturing maize hybrids under stress and non-stress conditions in two regions.

Top 10 hybrids	AD	ASI	EH	PH	EPO	EPP	Grain yield (t ha^-1^)					
							Across	Non-stress	Heat	Low N	Disease	Managed drought
Early												
CZL1432/CZL1207//CZL16031	67.78	0.42	106.05	211.06	0.51	1.05	5.23	7.35	3.54	3.12	5.22	3.49
CZL1432/CZL1207//CML571	65.71	1.26	105.54	206.05	0.52	1.03	5.21	7.38	3.57	3.09	5.11	3.89
CML571/CML546//CZL15225-B	64.69	1.75	91.29	198.52	0.47	0.95	5.18	7.07	3.52	3.15	5.14	4.00
CML571/CZL0919//CZL15110	63.42	1.58	91.60	202.96	0.45	1.00	5.17	7.12	3.34	3.06	6.57	3.50
CML571/CML546//CZL15110	63.46	1.60	93.57	200.52	0.47	0.98	5.10	7.03	3.46	3.02	5.41	3.78
CML571/CZL1385//CZL15225-B	65.77	1.55	97.08	206.54	0.48	1.02	5.10	7.29	3.32	3.09	5.18	3.27
CZL1432/CZL1207//CZL16014	68.82	1.28	99.34	201.56	0.50	1.01	5.09	7.33	3.32	2.81	4.91	3.91
CZL1432/CZL1348//CML571	66.07	1.78	98.31	201.35	0.51	0.97	5.07	7.16	3.43	2.83	5.90	3.59
CML571/CZL0919//CZL16151	63.88	1.20	99.68	207.46	0.49	0.97	5.06	7.05	3.42	3.08	5.39	3.60
CML571/CML546//CZL15089	63.95	0.98	97.63	195.94	0.50	1.00	5.05	7.01	3.44	2.82	6.66	3.53
Intermediate												
CML566/CML539//CML571	65.30	1.72	204.26	100.88	0.50	0.99	5.03	7.23	3.16	2.84	4.64	3.40
CML566/CZL1462//CZL15085-B	71.54	0.95	213.48	110.03	0.52	0.97	4.80	6.87	2.90	2.38	4.79	4.00
CML566/CML539//CZL15181	70.30	1.53	203.26	99.36	0.50	0.96	4.74	6.76	2.55	2.56	5.07	3.93
CML566/CZL1462//CKL05024	68.68	1.26	202.25	99.10	0.50	0.94	4.73	7.09	2.69	2.44	3.94	3.42
CML566/CML543//CZL15115	67.39	1.19	190.91	96.90	0.52	1.00	4.70	6.53	2.09	2.86	3.76	4.42
CML566/CML539//CZL1423	68.52	1.10	202.80	100.23	0.50	0.98	4.69	6.71	3.11	2.53	4.06	3.69
CML566/CML543//CZL1207	69.20	0.89	208.86	102.87	0.51	0.99	4.65	6.74	2.49	2.54	3.92	3.86
CML566/CML539//CZL16114	68.14	1.42	208.56	104.53	0.51	0.97	4.65	6.73	2.83	2.55	4.67	3.46
CML566/CZL1462//CZL16066	71.45	0.83	204.23	103.66	0.52	0.95	4.62	6.55	2.31	2.62	4.23	4.10
CML566/CML539//CZL16031	68.30	1.15	213.38	105.33	0.52	1.01	4.59	6.42	2.62	2.75	4.93	3.58
Checks												
CZH15002	64.27	2.03	90.19	197.47	0.46	0.91	4.95	7.12	3.39	2.89	4.22	3.92
CZH15383	68.60	1.12	107.97	211.19	0.52	0.97	4.75	7.07	3.25	3.08	3.50	3.17
SC529	65.46	1.65	99.31	206.56	0.49	0.95	4.72	7.13	3.35	2.62	4.02	3.93
PHB30G19	67.97	1.87	106.13	212.99	0.51	0.96	4.66	6.99	3.28	2.62	5.96	2.95
SC301	63.08	1.47	88.72	194.77	0.46	0.91	4.00	6.41	3.20	2.35	3.38	3.24
SC727	72.20	0.92	218.61	111.75	0.51	0.96	4.85	6.89	3.31	3.02	3.96	3.36
CZH15383	69.11	1.29	211.19	104.28	0.51	0.98	4.66	6.60	2.64	2.89	4.04	3.47
CZH15064	71.72	0.52	211.14	109.20	0.53	1.02	4.64	6.76	2.94	2.91	3.77	3.26
SC529	65.86	1.77	206.90	97.85	0.49	0.94	4.40	6.57	2.93	2.59	3.41	4.06
SC719	69.12	1.57	220.33	116.67	0.53	0.91	4.34	6.60	2.65	2.52	4.31	3.07

AD, anthesis days; ASI, anthesis–silking interval; EH, ear height; PH, plant height; EPO ear position; EPP, ears per plant.

The top 10 performing intermediate-maturing hybrids ranged from 4.59 to 5.03 t ha^-1^ across environments. Hybrid CML566/CZL539//CML571 showed the highest mean yields across all environments (5.03 t ha^-1^) as well as under optimum (7.23 t ha^-1^) and heat-stress conditions (3.16 t ha^-1^). However, it recorded the lowest mean yield under managed drought conditions (3.40 t ha^-1^). The mean yields among the best checks ranged from 4.00 to 4.95 t ha^-1^. Check CZH15002 exhibited the highest mean yield across environments (4.95 t ha^-1^), under non-stress conditions (7.12 t ha^-1^) and heat stress (3.39 t ha^-1^). Check PHB30G19 recorded the highest mean yield under disease pressure (5.96 t ha^-1^) but the lowest under managed drought (2.95 t ha^-1^). Check SC727 showed the lowest mean yield across all environments (4.00 t ha^-1^), under optimum (6.41 t ha^-1^) and disease conditions (3.38 t ha^-1^). In addition, check SC719 had the lowest yield under low nitrogen conditions (2.52 t ha^-1^).

### 3.2 Principal component analysis

**Figure 2:**
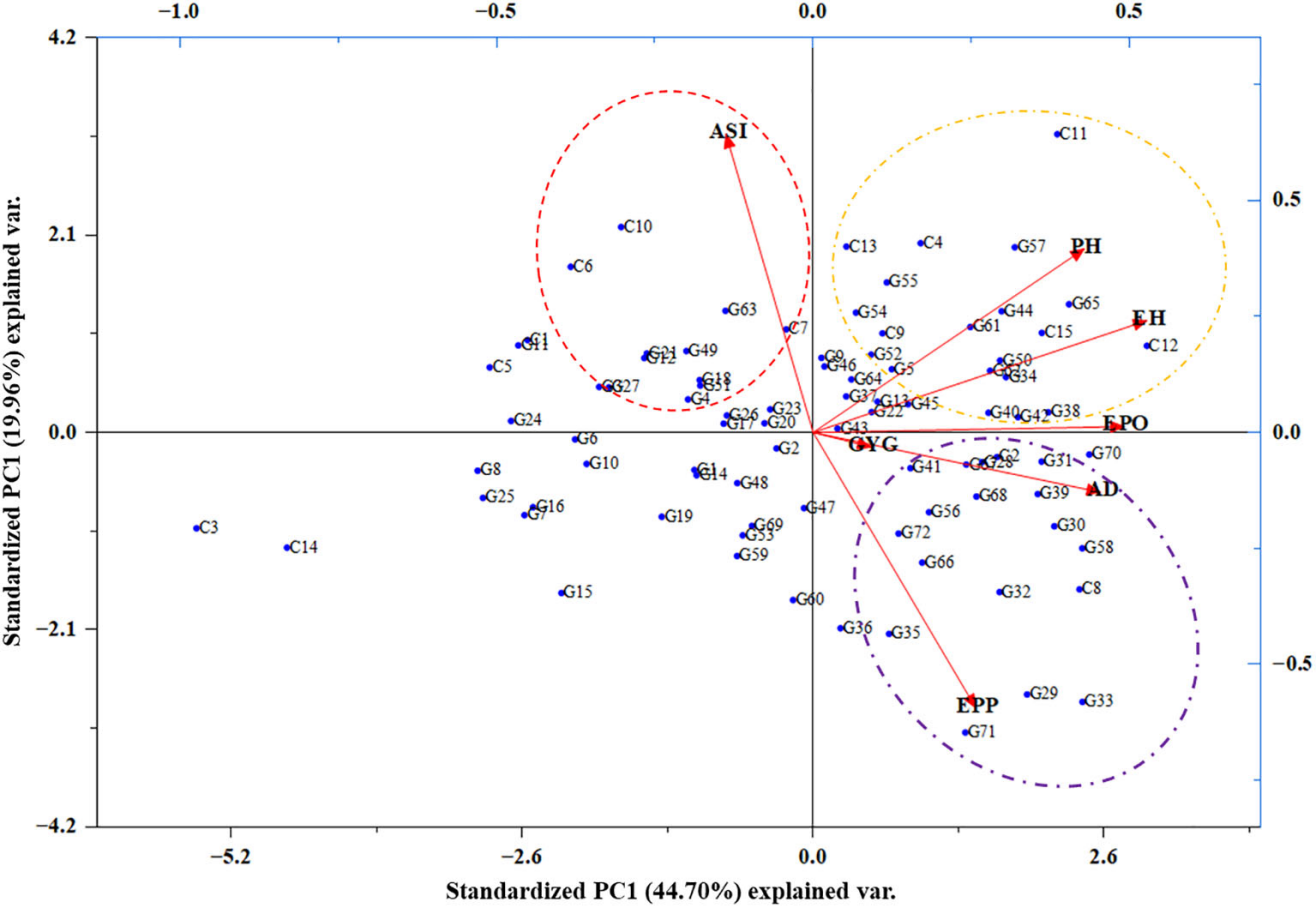
Genotype-by-trait PCA biplot showing the relationships among 87 maize hybrids and seven measured traits under non-stress environments based on the first two principal components, PC1 and PC2.

In the non-stress environments, three principal components (PC1, PC2, and PC3) had eigenvalues greater than 1, accounting for approximately 81.67% of the cumulative variation (**Table 4**). PC1 accounted for 44.70% of the total variance, whereas the ASI interval (-0,24) showed a negative effect. In contrast, AD (0.80), PH (0.76), EH (0.93), and EP (0.87) showed strong positive contributions. PC2 accounted for 19.96% of the variability, with ASI (0.79) and PH (0.47) showing strong positive loadings. PC3 accounted for 17.01% of the variability, with grain yield (0.95) and PH (0.25) exhibiting strong positive loadings. The PC biplot revealed that PH, EH, and EPO were closely grouped in the first quadrant (**Figure 2**). Anthesis days, grain yield, and EPP were also close together in the second quadrant. On the contrary, ASI in the fourth quadrant was distantly positioned relative to the other traits.

**Table 4:** Factor loadings, eigenvalue, percent, and cumulative variation.

	Non-stress			Heat			Low N		Disease		Managed drought	
	PC1	PC2	PC3	PC1	PC2	PC3	PC1	PC2	PC1	PC2	PC1	PC2
GYG	0.16	-0.04	0.95	0.82	-0.38	0.18	0.10	0.85	-0.27	0.85	0.23	0.85
AD	0.80	-0.15	-0.39	0.45	-0.42	-0.19	0.83	-0.08	0.64	-0.41	0.82	-0.18
ASI	-0.24	0.76	-0.13	-0.28	0.37	0.72	-0.66	0.11	0.12	0.37	-0.85	0.04
PH	0.76	0.47	0.25	0.44	0.49	-0.55	0.88	0.15	0.78	0.31	-0.74	0.10
EH	0.93	0.29	0.08	0.81	0.56	-0.02	0.96	0.10	0.95	0.28	0.86	-0.08
EPO	0.87	0.01	-0.20	0.72	0.40	0.30	0.87	0.03	0.88	0.15	0.94	-0.14
EPP	0.45	-0.70	0.09	0.73	-0.48	0.25	-0.26	0.75	-0.27	0.73	0.34	0.79
Eigenvalue	3.13	1.40	1.19	2.86	1.41	1.04	3.67	1.34	2.84	1.75	3.73	1.40
Variability (%)	44.70	19.96	17.01	40.85	20.16	14.80	52.40	19.20	40.59	25.02	53.25	20.06
Cumulative (%)	44.70	64.66	81.67	40.85	61.01	75.81	52.40	71.60`	40.59	65.62	53.25	73.31

PC, principal components; GYG, grain yield; AD, anthesis days; ASI, anthesis–silking interval; PH, plant height; EH, ear height; EPO, ear position; EPP, ears per plants.

**Figure 3:**
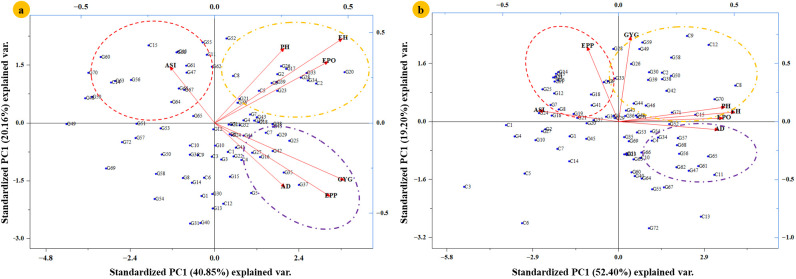
Genotype-by-trait PCA biplots showing the relationships among 87 maize hybrids and seven measured traits under **(a)** heat stress and **(b)** low-nitrogen environments based on the first two principal components, PC1 and PC2.

In the heat environments, three PCs (1, 2, and 3) had eigenvalues greater than 1, accounting for approximately 75.81% of the cumulative variation observed among the hybrids (**Table 4**). The contribution of PC1 was 40.85% of the total divergence, where grain yield (0.82), EH (0.81), EPP (0.73), and EPO (0.72) had strong positive contributions. In contrast, ASI (**-**0.28) had negative effects. PC2 accounted for 20.16% of the variability, with EH (0.56), PH (0.49), and EP (0.40) showing strong positive loadings. PC3 accounted for 14.80% of the variability, with AD (0.72), EP (0.30) and, EPP (0.25) showing strong positive loadings. The biplot revealed trait grouping similar to that in the non-stress environments (**Figure 3a**).

In low-N environments, two PCs (1 and 2) had eigenvalues > 1, accounting for approximately 71.60% of the cumulative variance (**Table 4**). PC1 accounted for 52.40% of the total divergence, and AD (0.83), PH (0.88), EH (0.96), and EP (0.87) showed strong positive contributions. PC2 contributed 19.20% of the variability in grain yield (0.85) and EPP (*r* = 0.75), with strong positive contributions. In contrast, AD (**-**0.08) showed a negative effect. The biplot revealed that PH, EH, and EP were closely grouped, whereas grain yield was not closely associated with the other traits (**Figure 3b**). The ASI and EPP were distantly positioned relative to the other traits and showed opposite trends.

**Figure 4:**
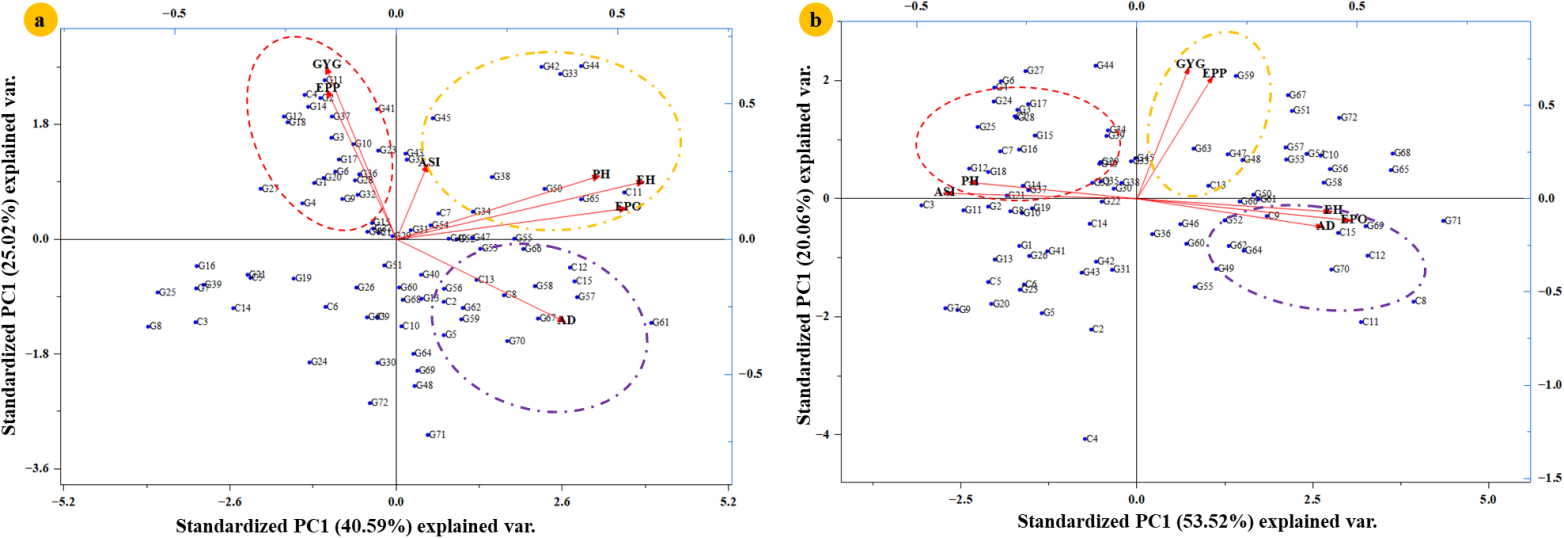
Genotype-by-trait biplot using PCA explaining the relationship between PC1 and PC2 for 87 hybrids and seven traits in **(a)** disease and **(b)** managed drought environment. .

In diseased environments, two PCs (1 and 2) had eigenvalues > 1, accounting for approximately 65.62% of the cumulative variation (**Table 4**). The contribution of PC1 was 40.59% of the total divergence, where anthesis days (0.64), plant height (0.78), ear height (0.95), and ear position (0.88) showed strong positive contributions. PC2 accounted for 25.02% of the variability, with grain yield (0.85) and ears per plant (0.73) exhibiting strong positive loadings. In contrast, anthesis days (**-**0.41) showed a negative effect. The biplot revealed that plant height, ear height, and ear position were closely grouped, whereas the ASI was separated from the other traits (**Figure 4a**). Conversely, grain yield, EPP, and AD were distantly positioned relative to the other traits.

In managed drought environments, two PCs (1 and 2) had eigenvalues >1, contributing approximately 73.31% of the cumulative variation (**Table 4**). PC1 accounted for 53.25% of the total divergence, with AD (0.82), EH (0.86), and EPO (0.94) showing strong positive contributions. PC2 accounted for 20.06% of the variability, with grain yield (0.85) and EPP (0.79) showing strong positive loadings. In contrast, AD (**-**0.18), EPO (**-**0.14), and EH (-0.08) showed negative effects. The biplot revealed that grain yield and EPP were closely grouped, while AD, EH, and EPO were closely grouped on the second quadrate. Conversely, ASI and PH were distantly positioned relative to the other traits and showed opposite trends (**Figure 4b**).

### 3.3 Correlations coefficient

**Figure 5:**
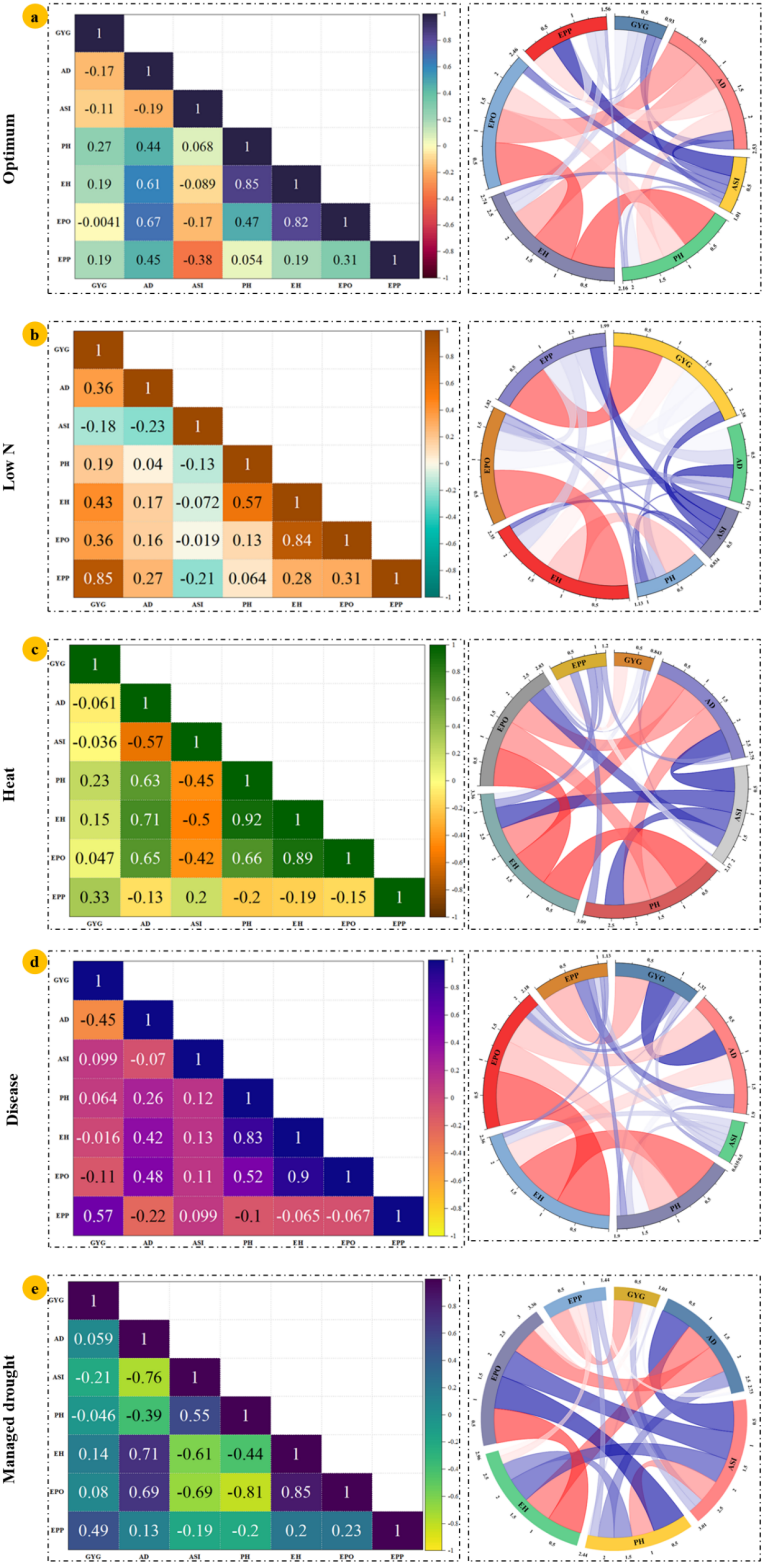
Correlation heatmaps and chord diagrams showing the relationships among seven agronomic traits of maize hybrids evaluated under different environments: **(a)** non-stress, **(b)** heat stress, **(c)** low nitrogen, **(d)** drought stress, and **(e)** combined stress conditions.

In non-stress environments, AD (*r* = -0.17), ASI (*r* = -0.11), and EPO (*r* = -0.041) showed negative correlations with grain yield (**Figure 5a**). Conversely, PH (*r* = 0.27), EH (*r* = 0.19), and EPP (*r* = 0.19) had a positive correlation with grain yield.

Across low N environments, anthesis days (*r* = -0.061) and anthesis–silking intervals (*r* = -0.036) showed a negative correlation with grain yield (**Figure 5b**). Conversely, plant height (*r* = 0.23), ear height (*r* = 0.15), ear position (*r* = 0.047), and ears per plant (*r* = 0.33) showed a positive correlation with grain yield.

In a heat environment, anthesis days (*r* = 0.36), plant height (*r* = 0.19), ear height (*r* = 0.43), ear position (*r* = 0.36), and ears per plant (*r* = 0.85) showed positive correlations with grain yield (**Figure 5c**). Conversely, anthesis–silking interval (*r* = -0.18) showed a negative correlation with grain yield.

In the diseased environment, anthesis–silking interval (*r* = 0.099), plant height (*r* = 0.064), and ears per plant (*r* = 0.56) showed strong positive correlations with grain yield (**Figure 5d**). Conversely, anthesis days (*r* = -0.45), ear height (*r* = -0.016), and ear position (*r* = -0.11) showed a negative correlation with grain yield.

In managed drought environments, anthesis–silking interval (*r* = -0.21) and plant height (*r* = -0.046) showed a negative correlation with grain yield (**Figure 5e**). Conversely, anthesis days (*r* = 0.059), ear height (*r* = 0.14), ear position (*r* = 0.08), and ears per plant (*r* = 0.49) showed a positive correlation with grain yield.

### 3.4 Path coefficient

The results of the path analysis showed the degree to which each independent variable under investigation was related to the grain yield. Path analysis was conducted separately for seven grain-yield-associated traits to partition correlation values into direct and indirect effects in stress and non-stress environments (**Table 5**). The residual effects varied across environments, ranging from 0.42 under heat stress to 0.82 under non-stress conditions, indicating that the included traits explained a substantial proportion of grain yield variation, particularly under stress environments. Under non-stress conditions, ear height (0.46) displayed a positive direct effect on grain yield and a negative indirect effect via ear position (-0.16). Ears per plant (0.40) had a direct effect on grain yield and a negative indirect effect on anthesis days (**-**0.27) and ear position (**-**0.07).

**Table 5:** The direct and indirect contribution of maize yield traits on grain yield under non-stress and stress environments.

Trait(s)	AD	ASI	PH	EH	EPO	EPP	GYG
Non-stress							
AD	-0.59	0.02	0.1	0.28	-0.16	0.18	-0.17
ASI	0.11	-0.09	0.02	-0.04	0.04	-0.15	-0.11
PH	-0.26	-0.01	0.23	0.39	-0.11	0.02	0.27
EH	-0.36	0.01	0.2	0.46	-0.19	0.08	0.19
EPO	-0.4	0.01	0.11	0.38	-0.23	0.12	0
EPP	-0.27	0.03	0.01	0.09	-0.07	0.4	0.19
COD (R2)							0.34
Residual effect (R)							0.82
Heat							
AD	0.12	-0.01	-0.01	0.11	-0.07	0.21	0.36
ASI	-0.03	0.03	0.02	-0.05	0.01	-0.16	-0.18
PH	0	0	-0.17	0.37	-0.05	0.05	0.19
EH	0.02	0	-0.1	0.64	-0.35	0.22	0.43
EPO	0.02	0	-0.02	0.54	-0.42	0.24	0.36
EPP	0.03	-0.01	-0.01	0.18	-0.16	0.78	0.85
COD (R2)							0.82
Residual effect (R)							0.42
Managed drought							
AD	-0.23	0.28	0.06	0.33	-0.45	0.06	0.06
ASI	0.17	-0.37	-0.09	-0.28	0.45	-0.09	-0.21
PH	0.09	-0.2	-0.16	-0.2	0.53	-0.09	-0.05
EH	-0.16	0.23	0.07	0.47	-0.56	0.09	0.14
EPO	-0.16	0.26	0.13	0.4	-0.66	0.11	0.08
EPP	-0.03	0.07	0.03	0.09	-0.15	0.47	0.49
COD (R2)							0.56
Residual effect (R)							0.66
Low N							
AD	-0.37	0.08	0.6	-0.52	0.21	-0.05	-0.06
ASI	0.21	-0.13	-0.43	0.37	-0.13	0.08	-0.04
PH	-0.23	0.06	0.95	-0.68	0.21	-0.08	0.23
EH	-0.26	0.07	0.87	-0.74	0.28	-0.08	0.15
EPO	-0.24	0.06	0.63	-0.66	0.32	-0.06	0.05
EPP	0.05	-0.03	-0.19	0.14	-0.05	0.41	0.33
COD (R2)							0.47
Residual effect (R)							0.73
Disease							
AD	-0.39	0	0.03	0.09	-0.07	-0.11	-0.45
ASI	0.03	0	0.01	0.03	-0.02	0.05	0.1
PH	-0.1	0	0.11	0.19	-0.07	-0.05	0.06
EH	-0.16	0	0.09	0.22	-0.13	-0.03	-0.02
EPO	-0.19	0	0.05	0.2	-0.14	-0.03	-0.11
EPP	0.09	0	-0.01	-0.01	0.01	0.5	0.57
COD (R2)							0.44
Residual effect (R)							0.75

COD, coefficient of determination; AD, anthesis days; ASI, anthesis–silking interval; PH,plant height; EH, ear height; EPO, ear position; EPP, ears per plants; GYG, grain yield.

In low-N environments, plant height (0.95) displayed a positive direct effect on grain yield and a negative indirect effect through ear height (**-**0.68) and anthesis days (**-**0.23). Ears per plant (0.41) displayed a positive direct effect on grain yield, and it also showed a negative indirect effect on plant height (**-**0.19), ear position (**-**0.05), and anthesis–silking interval (**-**0.03).

In heat-stress conditions, ears per plant (0.78) showed a positive direct effect on grain yield and a negative indirect effect on ear position (-0.16), plant height (-0.01), and anthesis–silking interval (**-**0.01). Ear height (0.64) showed a positive direct effect on grain yield and negative indirect effects on ear position (-0.35) and plant height (**-**0.10).

Disease stress conditions, ears per plant (0.50), showed a positive direct effect on grain yield and a negative indirect effect on plant height (**-**0.01) and ear height (**-**0.01). Ear height (0.22) showed a positive direct effect on grain yield and a negative indirect effect on anthesis days (-0.16), ear position (-0.13), and ears per plant (**-**0.03).

Under managed drought conditions, ear height (0.47) showed a positive direct effect on grain yield and a negative indirect effect on ear position (**-**0.56) and anthesis days (-0.16). Ears per plant (0.47) showed a positive direct effect on grain yield and a negative indirect effect on ear position (-0.15) and ears per plant (**-**0.03).

### 3.5 Cluster analysis

**Figure 6:**
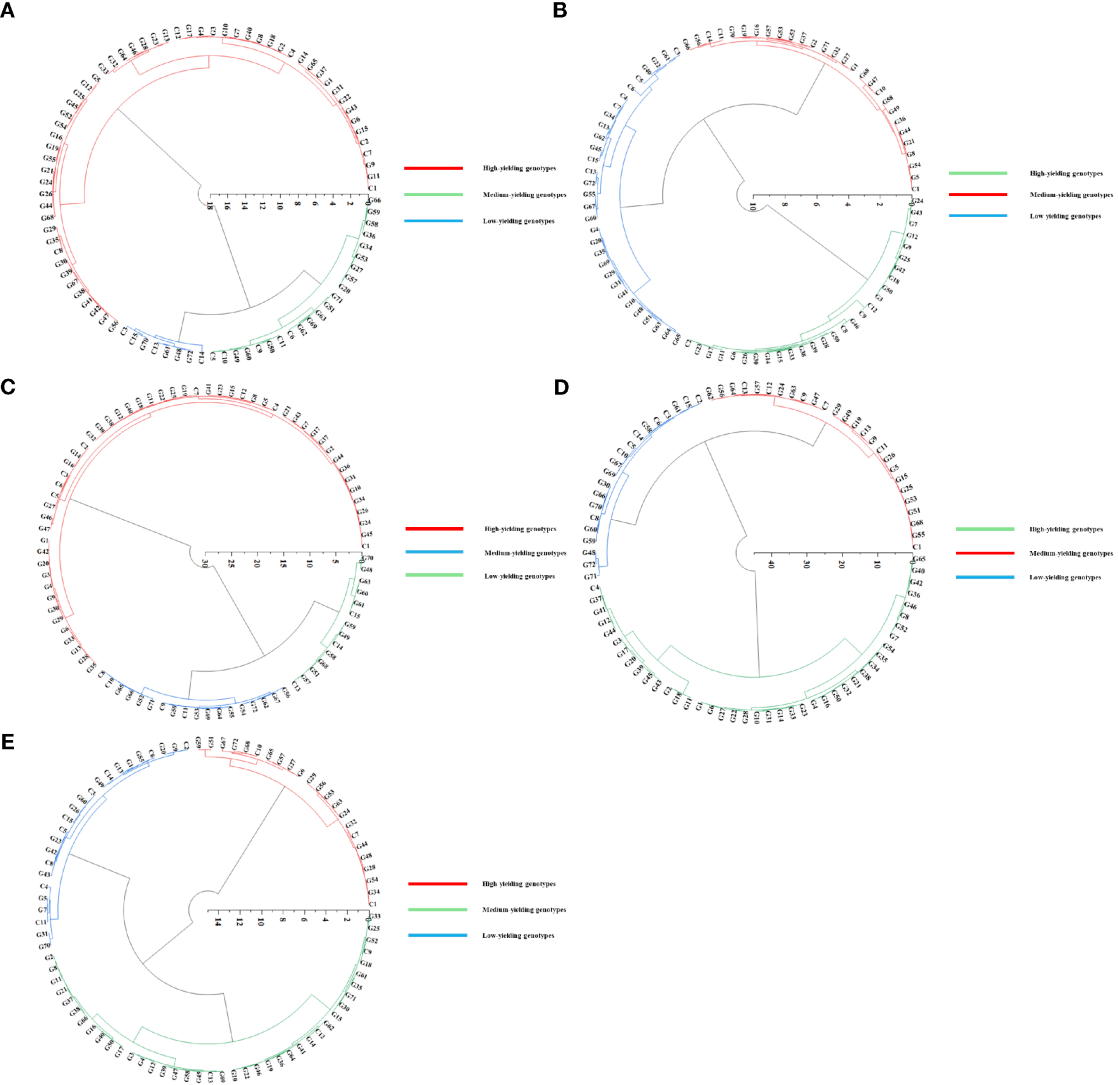
**(a)** Circular dendrogram showing the clustering pattern of maize genotypes based on grain yield performance under a non-stress environment. **(b)** Circular dendrogram showing the clustering pattern of maize genotypes based on grain yield performance under a low-N environment. **(c)** Circular dendrogram showing the clustering pattern of maize genotypes based on grain yield performance under a heat stress environment. **(d)** Circular dendrogram showing the clustering pattern of maize genotypes based on grain yield performance under a diseased environment. **(e)** Circular dendrogram showing the clustering pattern of maize genotypes based on grain yield performance under a managed drought environment.

Under the non-stress environment (**Figure 6a**), cluster analysis based on grain yield separated the evaluated genotypes into three major groups, indicating a variation in yield performance under favorable growing conditions. The largest cluster, shown in red, comprised most of the genotypes (57), suggesting that a large proportion of the materials had relatively similar yield responses under non-stress conditions. A second cluster, shown in green, grouped a smaller set of genotypes (22) that were distinct from the main cluster, indicating a different yield response pattern. The third cluster, shown in blue, contained the fewest genotypes (8) and was clearly separated from the other two groups, suggesting that these genotypes were the most divergent in terms of yield performance under the non-stress environment.

Under low-N conditions (**Figure 6b**), cluster analysis grouped the maize genotypes into three yield classes, reflecting variation in performance under nitrogen stress. The high-yielding cluster, shown in green, comprised 28 genotypes and represented the best-performing materials. The medium-yielding cluster, shown in red, also contained 28 genotypes with intermediate performance, while the low-yielding cluster, shown in blue, comprised 31 genotypes with the poorest yield response. The separation among clusters indicates substantial genotypic variation in grain yield under low-N conditions.

Under heat-stress conditions (**Figure 6c**), cluster analysis based on grain yield grouped maize genotypes into three yield classes, indicating differential responses to elevated temperature. The high-yielding cluster, shown in red, represented the largest group (55 genotypes), suggesting that most genotypes maintained relatively better yield performance under heat stress. The medium-yielding cluster, shown in blue, contained 18 genotypes with intermediate yield response, while the low-yielding cluster, shown in green, comprised 14 genotypes with the least performance. The separation of genotypes into distinct clusters demonstrates substantial variation in grain yield response under heat stress and highlights potential heat-tolerant genotypes for further evaluation.

Under diseased conditions (**Figure 6d**), cluster analysis based on grain yield grouped the maize genotypes, indicating variation in response to disease pressure. The high-yielding cluster, shown in green, represented the best-performing materials (41 genotypes) under disease stress. The medium-yielding cluster, shown in red, contained 26 genotypes with intermediate yield performance, while the low**-**yielding cluster, shown in blue, comprised 20 genotypes with the lowest yield response. The clear separation among clusters indicates substantial genotypic variation in grain yield under diseased conditions. It highlights the high-yielding genotypes as potential candidates for further evaluation and selection under disease-prone environments.

Under managed drought conditions, cluster analysis based on grain yield grouped the maize genotypes into three distinct yield classes, indicating variation in drought response (**Figure 6e**). The high-yielding cluster, shown in red, comprised 23 genotypes. The medium-yielding cluster, shown in green, contained 40 genotypes with intermediate yield performance, while the low-yielding cluster, shown in blue, comprised 24 genotypes with the poorest yield response. The separation of these clusters highlights substantial genotypic variation in grain yield under managed drought conditions. It identifies the high-yielding genotypes as potential candidates for further evaluation and selection in drought-prone environments.

## 4 Discussion

The observed significant genotype × environment interactions (GEI) for grain yield and associated traits under varying stress conditions show the complexity of breeding maize for stable performance across diverse environments. This is consistent with previous findings (Cairns et al., 2013; Prasanna et al., 2021) where it was highlighted that GEI is a major constraint in identifying broadly adapted genotypes under variable climatic conditions. However, including multiple stress factors in this study provides a more realistic representation of production conditions than studies that focus on single-stress environments. Similar to our results, these studies demonstrated that genotypes that perform well under non-stress conditions often fail to maintain superiority under stress, emphasizing the need for multi-environment testing.

However, the magnitude of GEI observed in this study appears higher than reported in some earlier regional trials, which may be attributed to the inclusion of diverse stress factors (heat, drought, low nitrogen, and disease) evaluated simultaneously, thereby increasing environmental heterogeneity. Prasanna et al. (2021) and Gbegbelegbe et al. (2024) demonstrated that integrating a multi-trait and multi-environment analysis can better capture the complexity of genotype responses to field-relevant changes, as emphasized in recent regional studies.

The superior performance of selected hybrids such as G6 (CML571/CML546//CZL15225-B) and G46 (CML571/CML566//CML571) under low N, heat, and managed drought environments aligns with the research findings from Masuka et al. (2017) and Tarekegne et al. (2023), who reported consistent genetic gains in stress-tolerant maize hybrids developed by CIMMYT. However, unlike these studies, which often emphasize single-stress tolerance, the present study demonstrated the importance of combined-stress tolerance, thereby explaining the broader adaptability observed. The results demonstrated that germplasm combining drought and low N tolerance significantly enhances yield stability in marginal environments. The consistency observed in the current study suggests that these hybrids possess adaptive traits that confer resilience across multiple stresses. However, unlike some reports that linked yield stability primarily to drought tolerance, our findings highlight the importance of combined tolerance mechanisms, which may explain the broader adaptability observed.

The positive association between grain yield and secondary traits such as plant height, ear height, and ears per plant agrees with previous studies (Amegbor et al., 2022; Matin et al., 2022), which showed that these traits contribute directly or indirectly to yield improvement. Recent studies further highlight ear-related traits as major determinants of yield (Kosgei et al., 2025; Ma et al., 2022; Yang et al., 2020), with strong genetic links among ear traits, kernel number, and grain yield (Wang et al., 2023). In this study, ears per plant consistently contributed to yield in both correlation and path analyses under stress, indicating that reproductive success and sink capacity are important for maintaining yield. This supports earlier findings that reproductive traits become increasingly important under stress, where efficient resource allocation is required (Noor et al., 2019; Ye et al., 2023).

A negative correlation between grain yield and anthesis–silking interval was observed across most environments, agreeing with Bänziger et al. (2000) and Cairns et al. (2013), who reported anthesis–silking interval as a reliable index of drought and heat tolerance. A shorter ASI reflects greater synchrony between male and female flowering, enhancing fertilization success under stress. While the present study confirms this widely reported trend, a slight positive or weak association under disease environments suggests that ASI may behave differently depending on the dominant stress factor. The weakening of the direct influence of reproductive timing on yield could explain this discrepancy.

The GT biplot analysis effectively revealed trait interactions and genotype performance across environments, supporting Yan and Rajcan (2002) and Lima et al. (2023), who demonstrated the use of biplots in visualizing complex trait associations. The clustering of ear height, ear position, and plant height observed in this study is consistent with previous reports (Kosgei et al., 2025; Li et al., 2025), indicating that these traits are structurally and genetically related. However, unlike some studies in which grain yield clusters closely with multiple traits, our results showed that grain yield was sometimes separated under low-N conditions. This discrepancy may be due to nutrient stress limiting biomass accumulation, thereby weakening the direct association between secondary traits and yield.

The identification of hybrids positioned close to the grain yield vector in the biplot confirms their superior performance and stability, in agreement with Amegbor et al. (2022), who noted that proximity to the yield vector indicates desirable multi-trait performance. The consistency of the patterns across stress environments in our study further validates the effectiveness of GT biplot analysis for selecting stable genotypes. The stronger discrimination observed under heat and low N stress than in non-stress environments suggests that stress conditions enhance phenotypic differentiation among genotypes, thereby making selection more efficient.

Cluster analysis revealed substantial genetic diversity among the evaluated hybrids, supporting Suwarno et al. (2014), who emphasized the importance of heterotic grouping for maximizing breeding efficiency. The identification of distinct clusters with specific trait advantages indicates the potential to exploit heterosis through targeted crosses. While previous studies have reported similar clustering patterns, the current study uniquely demonstrates cluster consistency across multiple stress environments, suggesting stable genetic divergence. This may explain the identification of 20 hybrids with consistent performance, highlighting their potential for advancement in breeding programs.

The path coefficient analysis further confirmed that ears per plant and ear height exert strong direct effects on grain yield, consistent with previous findings (Matin et al., 2022). However, the magnitude of these effects varied across environments, indicating that trait contributions are environment-specific—for instance, plant height exhibited a strong direct effect under low N but not under drought, suggesting differential physiological responses to nutrient versus water stress. This variability highlights the importance of environment-specific trait prioritization in breeding programs. Incorporating hybrids [G46 (CML566/CZL539//CML571) and G6 (CML57/CML546//CZL15225-B)] into the regional testing network could accelerate genetic gains for multiple-stress tolerance in SSA.

Overall, the study’s findings are broadly consistent with the existing literature but also reveal important variation. While previous studies often focused on single stressors, integrating multiple stressors into research provides a realistic representation of farmers’ conditions in SSA. The observed discrepancies, particularly in trait–yield relationships across environments, can be attributed to differences in stress type, intensity, and their interactions. These results reinforce the need for integrated breeding approaches that consider multiple stress factors simultaneously.

Importantly, this study demonstrated that combining multivariate tools such as genotype-by-trait biplots, path analysis, and cluster analysis provides a robust framework for identifying and selecting stable, high-performing hybrids. This aligns with recommendations by Prasanna et al. (2021) advocating for integrated analytical approaches in climate-resilient maize breeding. The approach used here not only improves selection accuracy but also enhances the understanding of trait interactions under complex stress scenarios.

This study confirms that secondary traits—particularly ears per plant, ear height, and anthesis–silking interval—are critical for improving maize productivity under stress conditions. The consistency of these findings with previous research validates their use as indirect selection criteria, and the observed differences highlight the importance of context-specific breeding strategies. The identified hybrids, therefore, represent valuable genetic resources for advancing maize production in stress-prone environments of Southern Africa.

## 5 Conclusion

This study demonstrated significant genetic variation among early- and intermediate-maturing maize hybrids for grain yield and secondary traits across stress and non-stress environments. The significant G × E interaction indicated that hybrid performance varied across stress types and environments. Grain yield was positively associated with ears per plant, ear height, and plant height, while anthesis–silking interval was generally negatively associated with yield. Path analysis identified ears per plant and ear height as key contributors to yield under stress. Genotype-by-trait biplots and cluster analysis effectively classified the hybrids into different yield groups, identifying CML571/CML546//CZL15225-B and CML566/CZL539//CML571 as promising multiple-stress-resilient hybrids for climate-resilient maize breeding.

## Data Availability

The original contributions presented in the study are included in the article/**Supplementary Material**. Further inquiries can be directed to the corresponding author.

## Supplementary material

Supplementary Table 1 List of experimental hybrids and checks for early maturing hybrids used in the study during the 2019/2020 season. Zim: Zimbabwe; CIMMYT: International Maize and Wheat Improvement Center.

Supplementary Table 2 Mean performance of early- and intermediate-maturing maize hybrids across stress and non-stress environments.

Supplementary Table 3 Summary statistics of non-stress and stress environments.
