# Supplementary material for: Performance of early- and intermediate-maturing maize hybrids for grain yield and secondary traits under multiple stress environments
Source: Front Plant Sci. 2026 Jul 24;17:1808200. doi: 10.3389/fpls.2026.1808200 (PMC13449069; doi:10.3389/fpls.2026.1808200)
Supplement: Supplementary file 1 [file Table1.docx]

**Supplementary tables**

**Supplementary Table 1**: List of experimental hybrids and checks for early maturing hybrids used in the study during 2019/20 season

| **Early hybrids** | | | |  | **Intermediate hybrids** | | | |
| --- | --- | --- | --- | --- | --- | --- | --- | --- |
| **Entries** | **Code** | **Genotypes** | **Origin** |  | **Entries** | **Code** | **Genotypes** | **Origin** |
| 1 | G1 | CML571/CML546//CML545 | CIMMYT-Zim |  | 1 | G46 | CML566/CML539//CML571 | CIMMYT-Zim |
| 2 | G2 | CML571/CML546//CZL15089 | CIMMYT-Zim |  | 2 | G47 | CML566/CML539//CZL1423 | CIMMYT-Zim |
| 3 | G3 | CML571/CML546//CZL15110 | CIMMYT-Zim |  | 3 | G48 | CML566/CML539//CZL15082 | CIMMYT-Zim |
| 4 | G4 | CML571/CML546//CZL15111 | CIMMYT-Zim |  | 4 | G49 | CML566/CML539//CZL16014 | CIMMYT-Zim |
| 5 | G5 | CML571/CML546//CZL15215 | CIMMYT-Zim |  | 5 | G50 | CML566/CML539//CZL16031 | CIMMYT-Zim |
| 6 | G6 | CML571/CML546//CZL15225-B | CIMMYT-Zim |  | 6 | G51 | CML566/CML539//CZL16111 | CIMMYT-Zim |
| 7 | G7 | CML571/CML546//CZL16145 | CIMMYT-Zim |  | 7 | G52 | CML566/CML539//CZL16114 | CIMMYT-Zim |
| 8 | G8 | CML571/CML546//CZL16146 | CIMMYT-Zim |  | 8 | G53 | CML566/CML539//CZL16132 | CIMMYT-Zim |
| 9 | G9 | CML571/CML546//CZL16151 | CIMMYT-Zim |  | 9 | G54 | CML566/CML539//CZL15181 | CIMMYT-Zim |
| 10 | G10 | CML571/CZL0919//CML545 | CIMMYT-Zim |  | 10 | G55 | CML566/CML543//CML589 | CIMMYT-Zim |
| 11 | G11 | CML571/CZL0919//CZL15110 | CIMMYT-Zim |  | 11 | G56 | CML566/CML543//CZL1207 | CIMMYT-Zim |
| 12 | G12 | CML571/CZL0919//CZL15111 | CIMMYT-Zim |  | 12 | G57 | CML566/CML543//CZL15006 | CIMMYT-Zim |
| 13 | G13 | CML571/CZL0919//CZL15215 | CIMMYT-Zim |  | 13 | G58 | CML566/CML543//CZL15035-B | CIMMYT-Zim |
| 14 | G14 | CML571/CZL0919//CZL15225-B | CIMMYT-Zim |  | 14 | G59 | CML566/CML543//CZL15115 | CIMMYT-Zim |
| 15 | G15 | CML571/CZL0919//CZL16145 | CIMMYT-Zim |  | 15 | G60 | CML566/CML543//CZL15123 | CIMMYT-Zim |
| 16 | G16 | CML571/CZL0919//CZL16146 | CIMMYT-Zim |  | 16 | G61 | CML566/CML543//CZL15215 | CIMMYT-Zim |
| 17 | G17 | CML571/CZL0919//CZL16151 | CIMMYT-Zim |  | 17 | G62 | CML566/CML543//CZL16090-B | CIMMYT-Zim |
| 18 | G18 | CML571/CZL0919//ZL15089 | CIMMYT-Zim |  | 18 | G63 | CML566/CML543//CZL16108 | CIMMYT-Zim |
| 19 | G19 | CML571/CZL1385//CML545 | CIMMYT-Zim |  | 19 | G64 | CML566/CZL1462//CKL05024 | CIMMYT-Zim |
| 20 | G20 | CML571/CZL1385//CZL15089 | CIMMYT-Zim |  | 20 | G65 | CML566/CZL1462//CZL15085-B | CIMMYT-Zim |
| 21 | G21 | CML571/CZL1385//CZL15111 | CIMMYT-Zim |  | 21 | G66 | CML566/CZL1462//CZL15178 | CIMMYT-Zim |
| 22 | G22 | CML571/CZL1385//CZL15215 | CIMMYT-Zim |  | 22 | G67 | CML566/CZL1462//CZL16014 | CIMMYT-Zim |
| 23 | G23 | CML571/CZL1385//CZL15225-B | CIMMYT-Zim |  | 23 | G68 | CML566/CZL1462//CZL16066 | CIMMYT-Zim |
| 24 | G24 | CML571/CZL1385//CZL16145 | CIMMYT-Zim |  | 24 | G69 | CML566/CZL1462//CZL16111 | CIMMYT-Zim |
| 25 | G25 | CML571/CZL1385//CZL16146 | CIMMYT-Zim |  | 25 | G70 | CML566/CZL1462//CZL16113-B | CIMMYT-Zim |
| 26 | G26 | CML571/CZL1385//CZL16151 | CIMMYT-Zim |  | 26 | G71 | CML566/CZL1462//CZL16129 | CIMMYT-Zim |
| 27 | G19 | CML571/CZL1385//CML545 | CIMMYT-Zim |  | 27 | G72 | CML566/CZL1462//CZL16135-B | CIMMYT-Zim |
| 28 | G27 | CML571/CZL1385//ZL15110 | CIMMYT-Zim |  | 28 | C8 | CZH15064 | CIMMYT-Zim |
| 29 | G28 | CZL1432/CZL1207//CML571 | CIMMYT-Zim |  | 29 | C9 | CZH15383 | CIMMYT-Zim |
| 30 | G29 | CZL1432/CZL1207//CZL1423 | CIMMYT-Zim |  | 30 | C10 | SC529 | Seed Co |
| 31 | G30 | CZL1432/CZL1207//CZL15082 | CIMMYT-Zim |  | 31 | C11 | SC719 | Seed Co |
| 32 | G31 | CZL1432/CZL1207//CZL15181 | CIMMYT-Zim |  | 32 | C12 | SC727 | Seed Co |
| 33 | G32 | CZL1432/CZL1207//CZL16014 | CIMMYT-Zim |  | 33 | C13 | PHB30G19 | CORTEVA |
| 34 | G33 | CZL1432/CZL1207//CZL16031 | CIMMYT-Zim |  | 34 | C14 | PAN53 | CORTEVA |
| 35 | G34 | CZL1432/CZL1207//CZL16114 | CIMMYT-Zim |  | 35 | C15 | PAN7M-81 | CORTEVA |
| 36 | G35 | CZL1432/CZL1207//CZL16132 | CIMMYT-Zim |  |  |  |  |  |
| 37 | G36 | CZL1432/CZL1207//ZL16111 | CIMMYT-Zim |  |  |  |  |  |
| 38 | G37 | CZL1432/CZL1348//CML571 | CIMMYT-Zim |  |  |  |  |  |
| 39 | G38 | CZL1432/CZL1348//CZL1423 | CIMMYT-Zim |  |  |  |  |  |
| 40 | G39 | CZL1432/CZL1348//CZL15082 | CIMMYT-Zim |  |  |  |  |  |
| 41 | G40 | CZL1432/CZL1348//CZL15181 | CIMMYT-Zim |  |  |  |  |  |
| 42 | G41 | CZL1432/CZL1348//CZL16014 | CIMMYT-Zim |  |  |  |  |  |
| 43 | G42 | CZL1432/CZL1348//CZL16031 | CIMMYT-Zim |  |  |  |  |  |
| 44 | G43 | CZL1432/CZL1348//CZL16111 | CIMMYT-Zim |  |  |  |  |  |
| 45 | G44 | CZL1432/CZL1348//CZL16114 | CIMMYT-Zim |  |  |  |  |  |
| 46 | G45 | CZL1432/CZL1348//CZL16132 | CIMMYT-Zim |  |  |  |  |  |
| 47 | C1 | CZH15002 | CIMMYT-Zim |  |  |  |  |  |
| 48 | C2 | CZH15383 | CIMMYT-Zim |  |  |  |  |  |
| 49 | C5 | SC301 | Seed Co |  |  |  |  |  |
| 50 | C6 | SC403 | Seed Co |  |  |  |  |  |
| 51 | C7 | SC529 | Seed Co |  |  |  |  |  |
| 52 | C4 | PHB30G19 | CORTEVA |  |  |  |  |  |
| 53 | C3 | PAN53 | CORTEVA |  |  |  |  |  |

**^Zim^**^: Zimbabwe;^ **^CIMMYT^**^: International Maize and Wheat Improvement Center^
