# Supplementary material for: Performance of early- and intermediate-maturing maize hybrids for grain yield and secondary traits under multiple stress environments
Source: Front Plant Sci. 2026 Jul 24;17:1808200. doi: 10.3389/fpls.2026.1808200 (PMC13449069; doi:10.3389/fpls.2026.1808200)
Supplement: Supplementary file 2 [file Table2.docx]

**Supplementary Table 2**: Mean performance of early and intermediate maturing maize hybrids across stress and non-stress enviroments

|  | **Environments** | | | | | |
| --- | --- | --- | --- | --- | --- | --- |
|  | **Across** | **Non-stress** | **Low N** | **Disease** | **Heat** | **Managed drought** |
| **Early hybrids** |  |  |  |  |  |  |
| CML571/CML546//CML545 | 4.97 | 7.19 | 2.77 | 5.17 | 3.48 | 3.17 |
| CML571/CML546//CZL15089 | 5.04 | 7.01 | 2.82 | 6.66 | 3.44 | 3.53 |
| CML571/CML546//CZL15110 | 5.11 | 7.03 | 3.02 | 5.41 | 3.46 | 3.78 |
| CML571/CML546//CZL15111 | 5.00 | 7.04 | 2.70 | 4.98 | 3.46 | 3.73 |
| CML571/CML546//CZL15215 | 4.74 | 6.95 | 2.90 | 4.23 | 3.28 | 3.08 |
| CML571/CML546//CZL15225-B | 5.18 | 7.07 | 3.15 | 5.14 | 3.52 | 4.00 |
| CML571/CML546//CZL16145 | 4.93 | 7.04 | 2.97 | 4.67 | 3.42 | 3.04 |
| CML571/CML546//CZL16146 | 4.93 | 7.00 | 2.88 | 4.64 | 3.29 | 3.55 |
| CML571/CML546//CZL16151 | 4.99 | 7.10 | 3.05 | 4.55 | 3.48 | 3.17 |
| CML571/CZL0919//CML545 | 4.90 | 7.02 | 2.67 | 5.30 | 3.38 | 3.39 |
| CML571/CZL0919//CZL15110 | 5.17 | 7.12 | 3.06 | 6.57 | 3.34 | 3.50 |
| CML571/CZL0919//CZL15111 | 4.97 | 6.94 | 3.03 | 5.71 | 3.33 | 3.74 |
| CML571/CZL0919//CZL15215 | 4.90 | 7.34 | 2.61 | 4.47 | 3.56 | 3.19 |
| CML571/CZL0919//CZL15225-B | 5.02 | 7.20 | 3.11 | 5.23 | 3.26 | 3.36 |
| CML571/CZL0919//CZL16145 | 5.01 | 7.13 | 3.12 | 4.17 | 3.32 | 3.45 |
| CML571/CZL0919//CZL16146 | 4.78 | 6.80 | 2.87 | 4.99 | 3.26 | 3.63 |
| CML571/CZL0919//CZL16151 | 5.07 | 7.05 | 3.08 | 5.39 | 3.42 | 3.60 |
| CML571/CZL0919//ZL15089 | 5.03 | 7.01 | 3.02 | 6.67 | 3.35 | 3.49 |
| CML571/CZL1385//CML545 | 4.71 | 6.82 | 2.86 | 4.37 | 3.36 | 3.44 |
| CML571/CZL1385//CZL15089 | 4.65 | 6.62 | 2.73 | 5.46 | 3.48 | 3.17 |
| CML571/CZL1385//CZL15111 | 4.83 | 6.85 | 2.89 | 5.02 | 3.43 | 3.57 |
| CML571/CZL1385//CZL15215 | 4.76 | 7.09 | 2.52 | 5.11 | 3.38 | 3.42 |
| CML571/CZL1385//CZL15225-B | 5.10 | 7.29 | 3.09 | 5.18 | 3.32 | 3.27 |
| CML571/CZL1385//CZL16145 | 4.80 | 6.84 | 2.95 | 4.00 | 3.39 | 3.83 |
| CML571/CZL1385//CZL16146 | 4.92 | 6.91 | 3.01 | 4.27 | 3.36 | 3.50 |
| CML571/CZL1385//CZL16151 | 4.84 | 6.82 | 3.14 | 4.31 | 3.39 | 3.23 |
| CML571/CZL1385//ZL15110 | 4.65 | 6.64 | 2.79 | 5.15 | 3.21 | 4.02 |
| CZL1432/CZL1207//CML571 | 5.21 | 7.38 | 3.09 | 5.11 | 3.57 | 3.89 |
| CZL1432/CZL1207//CZL1423 | 4.85 | 6.94 | 2.73 | 4.37 | 3.50 | 3.84 |
| CZL1432/CZL1207//CZL15082 | 4.87 | 6.94 | 3.15 | 3.60 | 3.48 | 3.43 |
| CZL1432/CZL1207//CZL15181 | 4.86 | 7.11 | 2.72 | 5.27 | 3.38 | 2.93 |
| CZL1432/CZL1207//CZL16014 | 5.09 | 7.33 | 2.81 | 4.91 | 3.32 | 3.91 |
| CZL1432/CZL1207//CZL16031 | 5.23 | 7.35 | 3.12 | 5.22 | 3.54 | 3.49 |
| CZL1432/CZL1207//CZL16114 | 4.57 | 6.59 | 2.60 | 5.05 | 3.40 | 3.92 |
| CZL1432/CZL1207//CZL16132 | 4.80 | 6.88 | 2.72 | 5.05 | 3.56 | 3.40 |
| CZL1432/CZL1207//ZL16111 | 4.76 | 6.75 | 2.89 | 4.73 | 3.39 | 3.42 |
| CZL1432/CZL1348//CML571 | 5.07 | 7.16 | 2.83 | 5.9 | 3.43 | 3.59 |
| CZL1432/CZL1348//CZL1423 | 4.96 | 6.95 | 3.13 | 5.03 | 3.34 | 3.55 |
| CZL1432/CZL1348//CZL15082 | 4.94 | 6.95 | 3.11 | 5.58 | 3.35 | 3.68 |
| CZL1432/CZL1348//CZL15181 | 4.70 | 7.00 | 2.49 | 4.86 | 3.34 | 3.61 |
| CZL1432/CZL1348//CZL16014 | 4.84 | 6.96 | 2.75 | 5.83 | 3.30 | 3.40 |
| CZL1432/CZL1348//CZL16031 | 4.88 | 6.97 | 3.02 | 4.72 | 3.47 | 3.28 |
| CZL1432/CZL1348//CZL16111 | 4.91 | 7.07 | 2.97 | 5.60 | 3.41 | 3.33 |
| CZL1432/CZL1348//CZL16114 | 4.88 | 6.83 | 2.89 | 5.77 | 3.37 | 3.90 |
| CZL1432/CZL1348//CZL16132 | 4.78 | 6.92 | 2.59 | 5.59 | 3.37 | 3.71 |
| CZH15002 | 4.95 | 7.12 | 2.89 | 4.22 | 3.39 | 3.92 |
| CZH15383 | 4.75 | 7.07 | 3.08 | 3.50 | 3.25 | 3.17 |
| PAN53 | 3.78 | 5.78 | 2.45 | 3.49 | 3.22 | 3.22 |
| PHB30G19 | 4.66 | 6.99 | 2.62 | 5.96 | 3.28 | 2.95 |
| SC301 | 3.98 | 6.41 | 2.35 | 3.38 | 3.20 | 3.24 |
| SC403 | 3.78 | 6.32 | 2.11 | 3.56 | 3.22 | 3.12 |
| SC529 | 4.72 | 7.13 | 2.62 | 4.02 | 3.35 | 3.93 |
| **Intermediate hybrids** |  |  |  |  |  |  |
| CML566/CML539//CML571 | 4.84 | 7.39 | 3.17 | 4.64 | 3.16 | 3.40 |
| CML566/CML539//CZL1423 | 4.61 | 6.96 | 2.88 | 4.06 | 3.11 | 3.69 |
| CML566/CML539//CZL15082 | 3.91 | 6.07 | 2.69 | 2.90 | 1.41 | 3.88 |
| CML566/CML539//CZL16014 | 4.15 | 6.47 | 2.90 | 4.38 | 2.14 | 3.12 |
| CML566/CML539//CZL16031 | 4.48 | 6.51 | 3.02 | 4.93 | 2.62 | 3.58 |
| CML566/CML539//CZL16111 | 4.21 | 6.22 | 2.67 | 4.20 | 2.26 | 4.31 |
| CML566/CML539//CZL16114 | 4.45 | 6.88 | 2.84 | 4.67 | 2.83 | 3.46 |
| CML566/CML539//CZL16132 | 4.48 | 6.65 | 2.86 | 4.23 | 2.71 | 3.85 |
| CML566/CML539//ZL15181 | 4.63 | 6.87 | 2.90 | 5.07 | 2.55 | 3.93 |
| CML566/CML543//CML589 | 4.30 | 6.83 | 2.55 | 4.22 | 2.75 | 3.14 |
| CML566/CML543//CZL1207 | 4.58 | 7.01 | 2.80 | 3.92 | 2.49 | 3.86 |
| CML566/CML543//CZL15006 | 4.35 | 6.63 | 2.82 | 3.99 | 2.25 | 3.97 |
| CML566/CML543//CZL15035-B | 4.32 | 6.64 | 2.92 | 3.54 | 2.30 | 3.69 |
| CML566/CML543//CZL15115 | 4.49 | 6.66 | 3.15 | 3.76 | 2.09 | 4.42 |
| CML566/CML543//CZL15123 | 4.13 | 6.44 | 2.55 | 3.75 | 1.96 | 3.23 |
| CML566/CML543//CZL15215 | 3.89 | 6.12 | 2.42 | 3.51 | 1.87 | 3.40 |
| CML566/CML543//CZL16090-B | 4.09 | 6.31 | 2.59 | 3.89 | 2.59 | 3.35 |
| CML566/CML543//CZL16108 | 4.10 | 6.37 | 2.66 | 4.01 | 1.98 | 3.84 |
| CML566/CZL1462//CKL05024 | 4.71 | 7.43 | 2.69 | 3.94 | 2.69 | 3.42 |
| CML566/CZL1462//CZL15085-B | 4.55 | 7.22 | 2.72 | 4.79 | 2.90 | 4.00 |
| CML566/CZL1462//CZL15178 | 4.31 | 6.70 | 2.80 | 3.67 | 2.87 | 3.53 |
| CML566/CZL1462//CZL16014 | 4.39 | 6.94 | 2.57 | 3.39 | 2.59 | 4.20 |
| CML566/CZL1462//CZL16066 | 4.53 | 6.75 | 2.9 | 4.23 | 2.31 | 4.10 |
| CML566/CZL1462//CZL16111 | 4.17 | 6.31 | 2.71 | 3.39 | 2.72 | 3.69 |
| CML566/CZL1462//CZL16113-B | 3.93 | 5.95 | 2.84 | 3.68 | 1.57 | 2.95 |
| CML566/CZL1462//CZL16129 | 3.94 | 6.24 | 2.79 | 2.38 | 3.00 | 3.43 |
| CML566/CZL1462//CZL16135-B | 3.92 | 6.03 | 2.54 | 2.89 | 2.54 | 4.07 |
| CZH15064 | 4.63 | 6.89 | 3.22 | 3.77 | 2.94 | 3.26 |
| CZH15383 | 4.58 | 6.53 | 3.35 | 4.04 | 2.64 | 3.47 |
| SC529 | 4.36 | 6.43 | 2.89 | 3.41 | 2.93 | 4.06 |
| SC719 | 4.21 | 6.52 | 2.77 | 4.31 | 2.65 | 3.07 |
| SC727 | 4.75 | 7.04 | 3.36 | 3.96 | 3.31 | 3.36 |
| PHB30G19 | 3.99 | 6.12 | 2.56 | 3.94 | 2.28 | 3.67 |
| PAN53 | 3.77 | 5.50 | 2.79 | 3.56 | 2.19 | 3.12 |
| PAN7M-81 | 3.91 | 5.90 | 2.58 | 3.50 | 1.92 | 3.27 |
