# Supplementary material for: Performance of early- and intermediate-maturing maize hybrids for grain yield and secondary traits under multiple stress environments
Source: Front Plant Sci. 2026 Jul 24;17:1808200. doi: 10.3389/fpls.2026.1808200 (PMC13449069; doi:10.3389/fpls.2026.1808200)
Supplement: Supplementary file 3 [file Table3.docx]

**Supplementary Table 3**: Summary statistics of non-stress and stress environments

| **Non-stress** | | | |  | **Low N** | | | |
| --- | --- | --- | --- | --- | --- | --- | --- | --- |
| **Minimum** | **Maximum** | **Mean** | **Std. deviation** |  | **Minimum** | **Maximum** | **Mean** | **Std. deviation** |
| 5.50 | 7.43 | 6.80 | 0.40 |  | 2.11 | 3.36 | 2.84 | 0.23 |
| 59.46 | 71.11 | 65.13 | 2.96 |  | 59.94 | 73.07 | 66.47 | 2.99 |
| 0.13 | 1.88 | 1.05 | 0.36 |  | 1.20 | 2.24 | 1.61 | 0.20 |
| 177.43 | 232.40 | 209.4 | 9.11 |  | 170.62 | 212.44 | 197.13 | 8.43 |
| 83.68 | 127.17 | 107.78 | 7.93 |  | 71.55 | 105.49 | 91.11 | 6.90 |
| 0.47 | 0.55 | 0.52 | 0.02 |  | 0.42 | 0.49 | 0.46 | 0.02 |
| 0.98 | 1.27 | 1.08 | 0.05 |  | 0.77 | 1.02 | 0.90 | 0.04 |
| **Heat** | | | |  | **Diseased** | | | |
| **Minimum** | **Maximum** | **Mean** | **Std. deviation** |  | **Minimum** | **Maximum** | **Mean** | **Std. deviation** |
| 1.41 | 3.57 | 3.02 | 0.53 |  | 2.38 | 6.67 | 4.53 | 0.87 |
| 58.19 | 67.81 | 61.89 | 1.44 |  | 73.22 | 85.29 | 78.63 | 3.04 |
| -2.50 | 3.00 | 0.41 | 1.20 |  | -1.00 | 2.00 | 0.58 | 0.54 |
| 129.5 | 172.47 | 150.13 | 9.82 |  | 189.77 | 231.66 | 213.21 | 8.60 |
| 66.52 | 85.51 | 76.36 | 3.95 |  | 94.52 | 139.05 | 116.79 | 9.35 |
| 0.47 | 0.57 | 0.51 | 0.02 |  | 0.48 | 0.61 | 0.55 | 0.03 |
| 0.62 | 0.85 | 0.77 | 0.05 |  | 0.83 | 1.10 | 0.96 | 0.05 |
| **Managed drought** | | | |  |  |  |  |  |
| **Minimum** | **Maximum** | **Mean** | **Std. deviation** |  |  |  |  |  |
| 2.93 | 4.42 | 3.55 | 0.33 |  |  |  |  |  |
| 82.86 | 92.58 | 87.55 | 2.11 |  |  |  |  |  |
| -0.30 | 2.62 | 1.91 | 0.60 |  |  |  |  |  |
| 145.17 | 181.30 | 166.68 | 9.60 |  |  |  |  |  |
| 54.29 | 104.48 | 81.16 | 9.92 |  |  |  |  |  |
| 0.38 | 0.65 | 0.49 | 0.08 |  |  |  |  |  |
| 0.69 | 0.95 | 0.86 | 0.04 |  |  |  |  |  |
